# Delayed anti-TNF therapy increases the risk of total knee replacement in patients with severe rheumatoid arthritis

**DOI:** 10.1186/s12891-017-1685-z

**Published:** 2017-08-01

**Authors:** Ying-Chou Chen, Wen-Chan Chiu, Tien-Tsai Cheng, Han-Ming Lai, Shan-Fu Yu, Ben Yu-Jih Su, Chung-Yuan Hsu, Chi-Hua Ko, Jia-Feng Chen

**Affiliations:** Department of Rheumatology, Kaohsiung Chang Gung Memorial Hospital, Chang Gung University College of Medicine, #123 Ta-Pei Road, Niao-Sung Dist, Kaohsiung, 833 Taiwan

**Keywords:** DAS28, Rheumatoid arthritis, RA, Anti-TNF therapy, Total knee replacement, TKR, Delayed intervention

## Abstract

**Background:**

This study evaluated the effect of early anti-tumor necrosis factor (TNF) therapy in patients with severe rheumatoid arthritis (RA) on the subsequent risk of total knee replacement (TKR) surgery.

**Methods:**

This retrospective observational study included a hospital-based cohort of 200 patients diagnosed with severe RA who received treatment with anti-TNF therapy between 2003 and 2014. Clinical parameters including age, sex, body mass index, and the time from the diagnosis of RA to the initiation of anti-TNF therapy were analyzed.

**Results:**

Of the 200 enrolled patients, 84 underwent an early intervention (≤3 years from the diagnosis of RA to the initiation of anti-TNF therapy), and 116 underwent a late intervention(>3 years from the diagnosis of RA to the initiation of anti-TNF therapy). Five (6.0%) patients in the early intervention group underwent TKR compared to 31 (26.7%) in the late intervention group (*p* = 0.023). After adjusting for confounding factors, the late intervention group still had a significantly higher risk of TKR (*p* = 0.004; odds ratio, 5.572; 95% confidence interval, 1.933–16.062). Those receiving treatment including methotrexate had a lower risk of TKR (*p* = 0.004; odds ratio, 0.287; 95% confidence interval, 0.122–0.672).

**Conclusions:**

Delayed initiation of anti-TNF therapy in the treatment of severe RA was associated with an increased risk of TKR surgery. Adding methotrexate treatment decreased the risk of future TKR.

## Background

Rheumatoid arthritis (RA) is a major public health problem and one of the most common auto-immune diseases. Patients with RA typically experience significant disability, reduced life expectancy, and poor quality of life [1]. As RA progresses, patients develop deformities and stiffness of the joints, especially in the hands and feet, and this is the major cause of disability. Damage to the joints is progressive and irreversible. The knee is a major weight-bearing joint in which patients with severe RA may experience bone destruction and disability [2–5], and some will require total knee replacement (TKR). Therefore, patients with RA can have significant long-term consequences [6–9]. One 18-year longitudinal study published in 1998 estimated that 25% of all patients with RA required total joint replacement surgery [10]. However, the incidence of joint replacement surgery in patients with RA has decreased in recent years, primarily because of the development of more effective medical treatment [11–13]. In a Norwegian register-based study, Nystad and Fenstad reported a decrease in the incidence of orthopedic surgery in patients with RA that continued into the era of biologics. The general increasing trend in the use of synthetic and biological disease-modifying anti-rheumatic drugs (DMARDs) has thus reduced the incidence of joint destruction and improved the long-term prognosis of patients with RA [14].

Current anti-rheumatic therapies attempt to alleviate pain and either delay or prevent joint deterioration. Traditional DMARDs such as methotrexate can control disease activity and prevent joint destruction in patients with RA [15]. Da Silva et al. reported a trend of a reduction in the incidence of joint surgery in patients with RA since 1985, due to advances in medical management [16]. In addition, Widdifield et al. reported that a longer duration of exposure to DMARDs soon after a diagnosis of RA was associated with a longer time to joint replacement surgery. Early intensive treatment for RA has also been reported to reduce the need of joint replacement surgery [17]. These findings have important implications for the utilization of health care resources.

Tumor necrosis factor (TNF) is known to play a major role in joint destruction in RA, and anti-TNF-α agents have been shown to slow the progression of joint destruction [18]. To date, however, there is little information regarding the effect of biological agents on the need for joint replacement surgery. Although Momohara and De Piano found a decrease in the incidence of joint replacement surgery after the use of biologics, Aaltonen and colleagues reported that the use of biological drugs did not reduce the need for joint replacement surgery in patients with similar on-medication disease activity [19].

The purpose of this study was to examine the effect of a delayed initiation of anti-TNF therapy in patients diagnosed with severe RA on the subsequent risk of TKR surgery. The hypothesis was that the delayed use of biological anti-rheumatic drugs would increase the need for joint replacement surgery due to progressive tissue damage caused by RA.

## Methods

This study was approved by the Institutional Review Board of Chang Gung Memorial Hospital in Kaohsiung, Taiwan, and it was conducted in accordance with the Good Clinical Practice Guidelines. All information was de-identified before data analysis, and thus informed consent was not required. Data of all patients with RA who received anti-TNF therapy between January 2003 and December 2014 were reviewed. The inclusion criteria were: 1) a diagnosis of RA based on the 1987 American College of Rheumatology criteria [20]; 2) severe RA (i.e., disease activity score > 5.1 based on the DAS28 criteria) before anti-TNF treatment for more than 6 months; and 3) taking at least two DMARDs for more than 2 years. The exclusion criteria were an age ≤ 20 years or >80 years and those who received TKR before the initiation of anti-TNF therapy.

Age, sex, body mass index (BMI), underlying diseases, use of concomitant DMARDs (i.e., hydroxychloroquine, methotrexate, leflunomide, cyclosporine, sulfasalazine, azathioprine), erythrocyte sedimentation rate, and C-reactive protein level were recorded at baseline and throughout the study period. DAS28 was calculated before anti-TNF therapy and every 3 months thereafter. The patients who underwent TKR during the 12-year study period were identified and analyzed. The time from the diagnosis of RA to the initiation of anti-TNF therapy was also analyzed. Follow-up data for each participant were recorded from the time of the initiation of anti-TNF therapy to November 30, 2014.

### Statistical Analysis

All statistical analyses were performed using SPSS software, version 21.0 (SPSS; Chicago, IL, USA). Logistic regression analysis was used to study the associations between various factors and TKR, and the odds ratios (ORs) for TKR in the patients with RA prescribed with anti-TNF therapy (years) were calculated, with adjustments for possible confounding factors such as DAS28 and the use of methotrexate.

## Results

A total of 1258 patients with RA were identified during the study period, of whom 256 had severe RA. Fifty of the patients with severe RA did not received biological anti-TNF agents and 206 did, six of whom were excluded due to undergoing TKR prior to anti-TNF agent therapy. Overall 200 patients with severe RA received anti-TNF agents (etanercept, 104 [52.0%]; adalimumab, 92 [46.0%]; and 4 (2%) golimumab. Among them, 84 underwent an early intervention (≤3 years from the diagnosis of RA to the initiating anti-TNF therapy), and 116 underwent a late intervention (>3 years from the diagnosis of RA to the initiation of anti-TNF therapy) (Table 1). The mean age at the diagnosis of RA in the early intervention group was 48.99 ± 13.46 years, which was significantly older than in the late intervention group (*p* = 0.002). In the early intervention group, 5 (6.0%) patients underwent TKR, compared to 31 (26.7%) in the late intervention group (*p* = 0.023).

**Table 1:** Baseline demographic and clinical characteristics of the patients with rheumatoid

Characteristic	Early intervention (*n* = 84)	Late intervention (*n* = 116)	*p* value
RA diagnosis age (years)	48.99 ± 13.46	43.68 ± 10.72	0.002
Sex (female, n %)	68 (81.0)	91 (78.4)	0.725
Body mass index (kg/m2)	22.60 ± 3.45	23.17 ± 3.63	0.267
Total knee replacement (n %)	5 (6.0)	30 (25.9)	0.023
Baseline DAS28 score (n %)	6.37 ± 1.1	6.42 ± 1.04	0.863
DAS28 score (n %) at 1 year	2.61 ± 0.44	2.84 ± 0.74	0.107
ESR mm/h	52.67 ± 29.13	45.99 ± 24.89	0.266
CRP mg/L	22.03 ± 19.39	23.17 ± 20.67	0.651
Rheumatoid factor (n %)	69 (82.1)	92 (79.1)	0.479
Anti-CCP (n %)	68 (80.9)	93 (80.1)	0.577
Baseline bone erosion at hand	15 (17)	22 (18.9)	0.778
RA duration (years)	2.68 ± 0.37	7.15 ± 4.18	0.001
Smoking (n %)	5 (6.0)	7 (6.0)	0.614
Alcohol consumption (n %)	4 (4.8)	6 (5.2)	0.584
Diabetes (n %)	10 (11.9)	5 (4.3)	0.058
Hypertension (n %)	23 (27.4)	29 (25.2)	0.746
Liver disease (n %)	15 (17.9)	16 (13.8)	0.437
Kidney disease (n %)	4 (1.2)1	5 (4.3)	0.404
Heart disease (n %)	5 (6.0)	7 (6.0)	0.614
Pulmonary disease (n %)	3 (3.6)	2 (1.7)	0.652
Use of other RA mediations			
Methotrexate (n %)	67 (79.8)	88 (75.9)	0.608
Hydroxychloroquine (n %)	41 (48.8)	58 (50.0)	0.887
Leflunomide (n %)	14 (16.7)	16 (13.8)	0.689
Cyclosporin (n %)	14 (16.7)	16 (13.8)	0.689
Sulfasalazine (n %)	9 (10.7)	17 (14.7)	0.524

Abbreviations: *RA* rheumatoid arthritis, *TKR* total knee replacement, *DAS28* disease activity score in 28 joints, *Anti-CCP* anti-citrullinated protein antibodies, *RF* Rheumatoid factor, *ESR* Erythrocyte sedimentation rate, *CRP* C-reactive protein.

Arthritis who received early (≤3 years) and late (>3 years) anti-TNF therapy (*n* = 200)

There were no differences in gender, BMI, underlying medical illnesses and oral disease modifying anti-rheumatic agents. After adjusting for confounding factors, the late intervention group still had a higher risk of TKR (*p* = 0.001; OR, 5.572; 95% CI, 1.933–16.062). Those receiving treatment including methotrexate also had a lower risk of TKR (*p* = 0.004; OR, 0.287; 95% CI, 0.122–0.672) (Table 2).

**Table 2:** Multivariate analysis of the risk of TKR with delayed anti-TNF treatment after adjusting for confounding factors

						95% CI for HR	
Variables	Regression coefficient	SE	Wald	*p* value	HR	Lower	Upper
Late/early intervention with biological therapy	2.652	0.922	8.27	0.004	8.323	2.256	30.696
RA duration	−0.05	0.08	0.391	0.532	0.951	0.813	1.113
Methotrexate (yes/no)	−1.086	0.446	5.917	0.015	0.338	0.141	0.81
Leflunomide (yes/no)	−2.158	1.228	3.083	0.079	0.116	0.01	1.283
Cyclosporine (yes/no)	−0.54	0.602	0.807	0.369	0.583	0.179	6.034
Sulfasalazine (yes/no)	0.731	0.544	1.803	0.179	2.077	0.715	6.034
RA diagnosis age	−0.104	0.021	0.425	0.515	0.986	0.946	1.028
sex (female/male)	−0.11	0.557	0.039	0.843	0.896	0.301	2.668
Body mass index (kg/m2)	0.017	0.059	0.086	0.769	1.018	0.906	1.143
Diabetes	−0.416	0.805	0.267	0.605	0.66	0.136	3.197

*HR* Hazard ratio, *CI* confidence interval, *SE* standard error, *TKR* total knee replacement, *RA* rheumatoid art hritis, *DAS28* disease activity score in 28 joints, *TNF* tumor necrosis factor

## Discussion

The aim of the present study was to identify factors that affected the need for knee replacement surgery in patients with RA. This results showed that use of methotrexate decreased the need for TKR in patients with RA, which is consistent with the findings of da Silva et al. who found that patients prescribed with synthetic DMARDs had better clinical outcomes (i.e., disease activity, functional capacity, radiographic score, and other clinical measures) than those who did not receiving synthetic DMARDs [16].

After adjusting for confounding factors, a longer duration from the diagnosis of RA to the initiation of anti-TNF therapy significantly increased the need for subsequent TKR. A possible reasons why the delayed use of anti-TNF therapy in patients with severe RA may increase the risk of TKR is that anti-TNF agents can reduce disease activity in patients with RA and either slow or completely halt the progression of joint erosion, even when there are persistent clinical signs of joint inflammation [21–24]. Further, anti-TNF agents have prolonged effects on the joints. In particular, a long-term, open-label trial on the safety and efficacy of DMARDs for the treatment of RA indicated that anti-TNF agents (but not other DMARDs) had sustained efficacy and favorable safety profiles even after 3 years of use [25].

There are several limitations to this study. This was a retrospective study with a relatively small sample size, and all data were collected from secondary sources (hospital medical records). As such, there may have been missing data, data collected by different observers, and disparity in the criteria used for different patients. A larger sample size is needed to confirm the finding that the early initiation of anti-TNF therapy can reduce the risk of TKR. In addition, a prospective study would not have the weaknesses inherent in a retrospective study. However, all available data were used in this single center cohort, which means the study design and sample size were the best available to us. In addition, a limitation of the retrospective nature of the study is that radiographs of the knees before anti-TNF treatment were not available, so the key to delaying TKR was dependent on the status of the knee at the time of presentation. In addition, the ability to control the disease with drug therapy will be limited.

## Conclusions

It is generally accepted that patients with severe RA should seek medical attention and treatment as soon as possible. Our results suggest that when patients with RA delay the initiation of anti-TNF therapy, they have an increased risk of subsequent TKR. Further investigations on this topic are warranted to provide further important information that may help guide decisions with regards resource allocation for patients with RA.

## Abbreviations

Anti-CCP: Anti-citrullinated protein antibodies
BMI: Body mass index
CI: Confidence interval
CRP: C-reactive protein
DAS28: Disease activity score in 28 joints
DMARDs: Disease-modifying anti-rheumatic drugs
ESR: Erythrocyte sedimentation rate
HR: Hazard ratio
OR: Odds ratios
RA: Rheumatoid arthritis
RF: Rheumatoid factor
SE: Standard error
TKR: Total knee replacement
TNF: Anti-tumor necrosis factor
